# 3D-printed scaffold loaded with baicalin exosomes promotes bone defect repair via mediating PRRX2 to alleviate inflammation

**DOI:** 10.1016/j.isci.2025.113565

**Published:** 2025-09-12

**Authors:** Haotian Zhu, Kai Cheng, Mingwei Tian, Yuanhao Peng, Yadi Zhang, Han Yan, Shaoxing Fan, Bo Shang, JiaYi Wu, Huanwen Ding, Naru Zhao

**Affiliations:** 1 School of Medicine, South China University of Technology, Guangzhou 510006, China; 2 The Guangzhou First People’s Hospital, Guangzhou 510180, Guangdong, China; 3 School of Materials Science and Engineering, South China University of Technology, Guangzhou 510006, China; 4 Guangdong Pharmaceutical University, Guangzhou 510006, Guangdong, China

**Keywords:** Biological sciences, Engineering

## Abstract

Chronic nonunion of bone defects remains a significant challenge in orthopedic treatment. Artificial bone graft materials are expected to solve this problem due to their suitable degradation rate and good osteoconductivity. However, ROS and inflammation within the defect environment are important causes of implant failure. Exosomes derived from different preconditioned bone mesenchymal stem cells (BMSCs) have shown great potential in the treatment of various diseases. Here, we developed a 3D-β-TCP@BA-BMSC-exos scaffold that loaded baicalin-pretreated BMSC exosomes (BA-BMSC-exos) on a 3D-printed β-tricalcium phosphate scaffold (3D-β-TCP) for bone defect repair. *In vitro* experiments showed that BA-BMSC-exos enhanced proliferation, migration, and tube formation in HUVECs, as well as inhibited inflammation via mediating PRRX2. Moreover, 3D-β-TCP scaffolds loaded with BA-BMSC-exos clearly alleviated inflammation and promoted angiogenesis in a calvarial defect rat model. This study suggests that 3D-β-TCP scaffolds combined with BA-BMSC-exos are a promising strategy to enhance bone repair and regeneration.

## Highlights

• Baicalin-pretreated BMSC exosomes suppress inflammation via PRRX2
• 3D-β-TCP scaffolds deliver BA-BMSC-exos to enhance angiogenesis and bone repair
• BA-BMSC-exos regulate endothelial function and promote osteogenesis *in vivo*

## Graphical abstract

**Figure:**
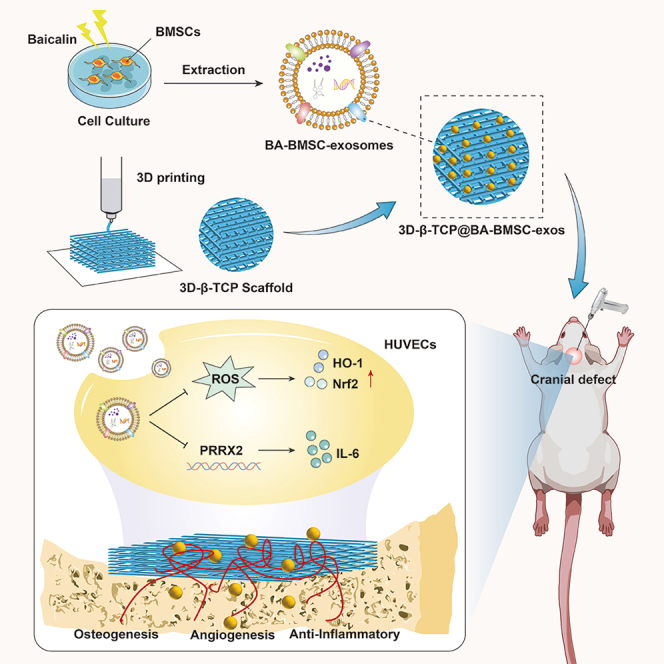

## Introduction

Bone defects caused by trauma, infection, tumor resection, or degenerative diseases remain a persistent clinical challenge. Current treatment options, such as autografts, allografts, and synthetic materials, each have inherent limitations. Autografts are osteoconductive and biocompatible but are restricted by donor site morbidity and limited availability.^1^ Allografts present risks including immune rejection, disease transmission, and slow remodeling.^2^ Synthetic materials often lack sufficient integration, angiogenesis, and mechanical strength.^3^ In addition, postoperative infection and chronic inflammation can further compromise graft survival and hinder bone regeneration. In the defect area, a series of pathological changes occur within the local microenvironment, among which the excessive accumulation of reactive oxygen species (ROS) serves as a central hallmark.^4^^,^^5^ Studies have demonstrated that hypoxia,^6^ inflammatory responses,^7^ and metabolic dysregulation^8^ following bone defects significantly elevate ROS production, disrupting intracellular redox equilibrium and inducing oxidative stress. In diabetic bone defects, hyperglycemic and hypoxic conditions promote macrophage polarization toward the pro-inflammatory M1 phenotype, triggering substantial ROS release and pro-inflammatory cytokine secretion (e.g., TNF-α, IL-6).^9^ These processes further suppress angiogenesis and osteogenic differentiation, thereby stalling tissue regeneration in a persistent inflammatory phase. Hence, there is a pressing need for a multifunctional bone tissue repair material that can not only rapidly remove ROS in the early stage, but also promote bone regeneration, which is essential for bone defect repair. Recently, advanced biomaterials and bioengineering approaches have garnered attention as alternative solutions for promoting bone regeneration and enhancing graft integration.^10^

Mesenchymal stem cells (MSCs), particularly those derived from bone marrow (BMSCs), have gained attention in regenerative medicine due to their potential to differentiate into various cell types, including osteoblasts, which are essential for bone formation.^11^^,^^12^ It has been reported that BMSCs exert their therapeutic effects through multiple mechanisms, including promoting neovascularization, inhibiting cell apoptosis, and mitigating inflammatory responses.^13^^,^^14^ Besides, multiple studies have shown that the therapeutic potential of BMSCs is largely attributed to their ability to secrete extracellular vesicles, particularly exosomes, which play a crucial role in cell-to-cell communication.^15^^,^^16^ BMSC-derived exosomes carry bioactive molecules such as proteins, lipids, and RNAs, which can influence the behavior of surrounding cells, thereby creating a pro-regenerative environment.^17^ Therefore, BMSCs-exos may be an ideal tool for the treatment of bone defects. However, the existence of ROS could directly damage cellular components, including DNA, lipids, and proteins, accelerating senescence and apoptosis of osteoblasts and BMSCs.^18^ Besides, it has been reported that MSCs-exos can lead to opposing effects during cartilage regeneration, including hypertrophic differentiation and subsequent calcification.^19^ To overcome these challenges, more effective and homogeneous exosomes should be explored.

Some studies have shown that preconditioning using cytokines, drugs, hypoxic conditions, or physical factors can improve the transplantation efficacy, thereby improving the biological function of BMSCs and enhancing the paracrine effect.^20^^,^^21^ Baicalin (BA), a flavonoid derived from the traditional Chinese herb Huang Qin (*Scutellaria baicalensis*), has been recognized for its anti-inflammatory, antioxidant, and pro-osteogenic properties.^22^^,^^23^ Studies have demonstrated that the protective effects of exosomes derived from baicalin-pretreated BMSCs can alleviate hepatic cell ferroptosis following acute liver injury,^24^ and improve Th17/Treg imbalance after hepatic ischemia–reperfusion.^25^ Therefore, it was also speculated that BA could stimulate BMSCs to produce potent exosomes to regulate chondrocyte metabolism and proliferation. However, the precise mechanisms of the action of Baicalin-pretreated BMSCs-derived exosomes (BA-exos) in bone defects remain unclear.

Three-dimensional (3D) printing technology has opened avenues in bone tissue engineering by allowing for the precise fabrication of customized scaffolds that mimic the natural bone structure.^26^^,^^27^ Calcium phosphate (CaP) bio-ceramics have been widely used for bone tissue repair, regeneration, and enhancement because of their characteristics of similar components to natural bones, good biocompatibility, biological activity, and non-immunogenicity.^28^ In particular, β-tricalcium phosphate (β-TCP), which is degradable and absorbable, can facilitate the adhesion and proliferation of preosteoblast cells and has attracted considerable attention.^29^ 3D-printed β-TCP scaffold serves as a biodegradable and osteoconductive carrier for the localized delivery of baicalin-preconditioned BMSC exosomes, providing structural support while facilitating sustained release of bioactive exosomes to enhance bone regeneration. Although previous studies have demonstrated the regenerative potential of 3D-printed β-TCP scaffolds and BMSC-derived exosomes separately,^30^^,^^31^ these approaches still face challenges related to poor bioactivity under inflammatory and oxidative stress conditions. Moreover, the heterogeneity of exosomes limits their consistent therapeutic efficacy. To address these issues, we propose a strategy by preconditioning BMSCs with BA to produce more functionally enhanced exosomes; meanwhile, the β-TCP scaffold was employed to serve as a biodegradable and osteoconductive carrier. This strategy not only ensures the sustained local delivery of bioactive exosomes but also provides a favorable microenvironment for bone regeneration.

In this study, we aim to investigate the effects of baicalin-preconditioned BMSC exosomes in combination with a 3D-printed β-TCP scaffold on bone defect repair, especially from the perspective of inducing angiogenesis. Through *in vitro* and *in vivo* experiments, we evaluated the angiogenic and osteogenic potential, as well as the underlying molecular mechanisms of this combined treatment, contributing to the development of advanced therapeutic strategies for bone tissue engineering.

## Results and discussion

### Preparation and characterization of 3D-β-tricalcium phosphate scaffolds

**Figure 1:**
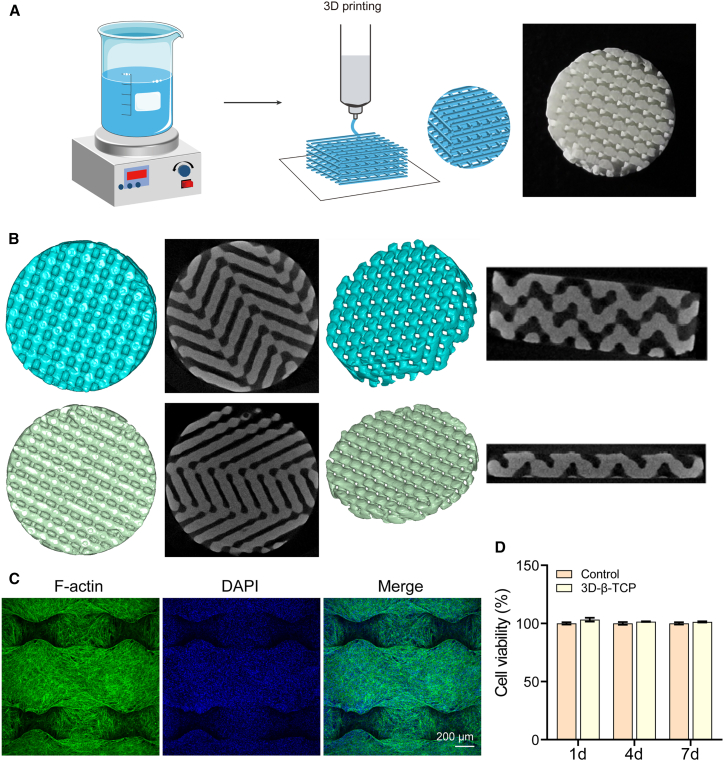
Characterization of 3D-β-TCP scaffold (A) Preparation process of 3D-TCP scaffold. (B) Three-dimensional reconstruction and Micro-CT images of the scaffold in cross-sectional and coronal views with different thickness. (C) F-actin images of 3D-TCP scaffold (scale bar: 200 μm). (D) Cell viability of the 3D-TCP scaffold at different times. Data are presented as mean ± standard deviation (SD), *n* = 3.

Two types of 3D-β-TCP scaffolds with different thicknesses were prepared as illustrated Figure 1A. The scaffold with 2 mm thickness was used for *in vitro* biocompatibility evaluation, and a 1 mm thickness scaffold was used for animal experiments to ensure better conformity to the anatomical structure of the skull and optimal integration with the defect site. All types of scaffolds were of the same diameter, 10 mm. Micro-CT was used to observe the precise geometry of the printed 3D-β-TCP scaffolds. As shown in Figure 1B, the cross-sectional and coronal views of the scaffolds and the 3D reconstruction models revealed the structural integrity and uniformity of the scaffolds. To assess the biocompatibility of 3D-β-TCP scaffolds, HUVECs were seeded onto the 3D-β-TCP scaffolds. F-actin staining was performed to visualize the cell cytoskeleton and the interaction between cells and the scaffold (Figure 1C). The results demonstrated that HUVECs adhered to the scaffold and formed a stable cell structure, indicating excellent biocompatibility and suitable surface properties for cell attachment. Additionally, CCK8 assay was conducted to assess the proliferation of HUVECs on the 3D-β-TCP scaffolds (Figure 1D). The results indicated significant cell growth on the scaffolds, with an increasing proliferation rate over time, confirming that the scaffolds provided a conducive environment for cell expansion. The enhanced cell viability suggests that the 3D-β-TCP scaffolds effectively support cellular proliferation, making them promising candidates for tissue engineering applications.

### Effect on angiogenesis of baicalin-pretreated bone mesenchymal stem cells exosomes--exos

**Figure 2:**
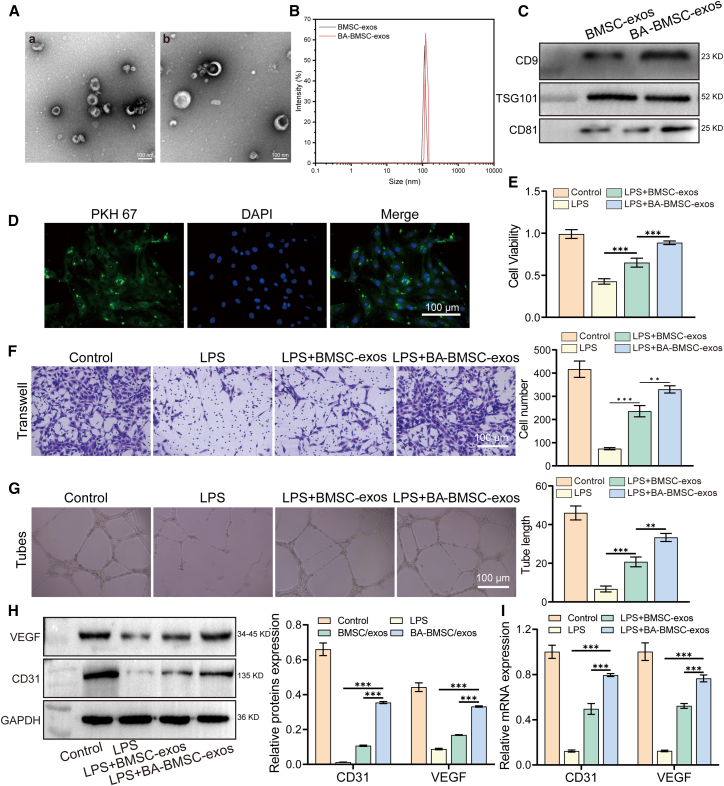
Identification and angiogenesis of exos from BA-pretreated BMSCs (A) The morphology of BMSC-exos and BA-BMSC-exos under transmission electron microscopy. (B) Nanoparticle tracking analysis showing the size distribution of BMSC-exos and BA-BMSC-exos. (C) The expression levels of the exosome markers CD9, TSG101, and CD81 were measured by western blot. (D) The uptake of BA-BMSC-exos by HUVECs was detected by immunofluorescence staining (scale bar: 100 μm). (E) CCK8 determined the viability of HUVECs after treatment with exos. (F) Cell migration of HUVECs determined by Transwell assay (scale bar: 100 μm). (G) Tube formation of HUVECs following treatment with exos (scale bar: 100 μm). The expression of VEGF and CD31 in HUVECs was determined by (H) Western blot and (I) qPCR. Data are presented as mean ± standard deviation (SD), *n* = 3, *p*-values are calculated using one-way or two-way ANOVA, ∗*p* < 0.05, ∗∗*p* < 0.01, ∗∗∗*p* < 0.001.

BA-BMSC-exos were isolated from the culture medium supernatant of baicalin pretreated BMSCs. The morphology of BA-BMSC-exos were observed by transmission electron microscopy (TEM). As shown in Figure 2A, the BMSC-exos and BA-BMSC-exos exhibited a cup-shaped or spherical structures. NTA results confirmed that the particle size distribution of BMSC-exos and BA-BMSC-exos falls within the range of 30–150 nm (Figure 2B). Western blot analysis confirmed that BA-BMSC-exos expressed the characteristic surface markers CD9, TSG101, and CD81 (Figure 2C). Moreover, we assessed the uptake ability of PKH 67 labeled-BA-BMSC-exos, and the results showed that BA-BMSC-exos were observed in HUVECs (Figure 2D). This result is consistent with the findings of Zhang et al.,^25^ demonstrating that exosomes derived from baicalin-treated BMSCs can be efficiently internalized by recipient cells.

To evaluate the effect of BA-BMSC-exos on the proliferation, migration, and tube formation of HUVECs. We first treated HUVECs with LPS to simulate the inflammatory model. Then, we assessed the cell proliferation by CCK8 and EdU assays. Figures 2E and S1 indicated that BA-BMSC-exos significantly enhanced cell proliferation of HUVECs compared to LPS and LPS+BMSC-exos groups. Cell migration and tube formation are two crucial factors of angiogenesis.^32^ Here, we examined the cell migration by the Transwell assay, which is a key step in angiogenesis. The results exhibited a significant increase in cell migration in the LPS+BA-BMSC-exos group compared to the LPS+BMSC-exos group (Figure 2F). Besides, Figure 2G showed that LPS-induced HUVECs treated with BA-BMSC-exos formed more and more structured tubular networks compared to other groups. These results were consistent with previous studies,^33^^,^^34^ suggesting that BA-BMSC-exos could enhance angiogenesis by regulating endothelial cell behavior and stimulating endothelial cell network formation, an essential feature of vascular development.

VEGF is a critical factor that promotes the proliferation and migration of vascular endothelial cells, and it can promote angiogenesis through the activation of the VEGFR signaling pathway.^35^ CD31, as a marker of endothelial cells, which reflects the density of blood vessels, participates in and indicates the degree of angiogenic activity together with VEGF.^36^ Here, we detected the expression of VEGF and CD31 in HUVECs through western blot, qPCR and immunofluorescence. As shown in Figures 2H and 2I, BA-BMSC-exos significantly upregulated the levels of VEGF and CD31 compared to LPS+BMSC-exos groups, suggesting that BA-BMSC-exos may enhance angiogenesis through the upregulation of angiogenesis-related signaling pathways and endothelial cell activation. Furthermore, immunofluorescence analysis also confirmed the similar results of BA-BMSC-exos (Figure S2).

### Baicalin-pretreated bone mesenchymal stem cells exosomes-exos inhibited inflammation via paired related homeobox 2/IL-6 pathway

**Figure 3:**
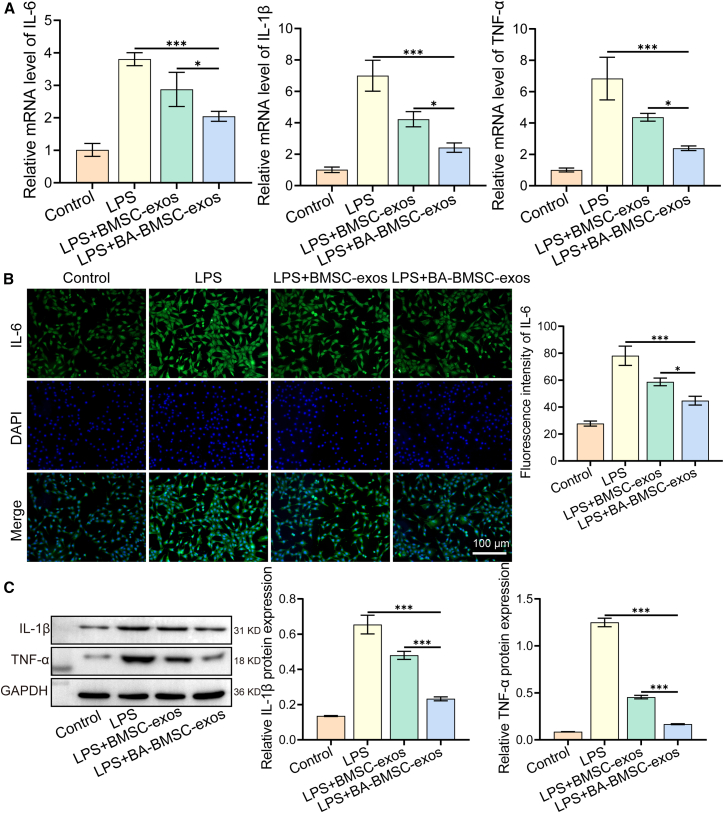
Anti-inflammation of BA-BMSC-exos (A) The expression levels of IL-1β, IL-6, and TNF-α in HUVECs determined by qPCR. (B) Immunofluorescence images of IL-6 in HUVECs (scale bar: 100 μm). (C) Western blot analysis of IL-1β and TNF-α in HUVECs. Data are presented as mean ± standard deviation (SD), *n* = 3, *p*-values are calculated using one-way or two-way ANOVA, ∗*p* < 0.05, ∗∗*p* < 0.01, ∗∗∗*p* < 0.001.

Bone defects can stimulate a series of immune and inflammatory responses that play a crucial role in the bone healing process.^37^ Inflammatory responses are necessary in the early stages of bone defects to help remove damaged tissue and initiate the healing process,^38^ but chronic or excessive inflammation can impede bone healing, leading to osteolysis and impaired repair.^39^ Here, we measured the expression levels of inflammatory cytokine (IL-6, IL-1β, and TNF-α) in HUVECs after 24 h treatment by qPCR, immunofluorescence staining, and western blot. qPCR analysis revealed that BA-BMSC-exos significantly reduced the expression of IL-6, IL-1β, and TNF-α compared to the LPS and LPS+BMSC-exos groups, indicating that BA-BMSC-exos have an anti-inflammatory effect (Figure 3A). Immunofluorescence and western blot analyses further confirmed a significant reduction of IL-6, IL-1β, and TNF-α in the LPS+BA-BMSC-exos group (Figures 3B and 3C). To evaluate the persistence of the anti-inflammatory effect, we further assessed cytokine levels at 48 h post-treatment by ELISA kit, which showed consistent downregulation trends of BA-BMSC-exos treatment (Figure S5A). These results suggest that BA-BMSC-exos could reduce sustained inflammatory signaling to maintain a balanced immune environment. Numerous studies have reported that the excessive production of ROS in bone defect microenvironments can lead to oxidative stress-induced damage.^40^^,^^41^ Therefore, we investigated the ROS-scavenging capability of BA-BMSC-exos. Flow cytometry analysis showed that BA-BMSC-exos significantly reduced the ROS level compared to LPS and LPS+BMSC-exos groups (Figure S3C). Moreover, researchers have found that the Nrf2/HO-1 signaling pathway plays a significant role in bone repair.^42^^,^^43^^,^^44^ Nrf2 is a well-known antioxidant transcription factor that responds to oxidative stress, safeguards cells by binding to antioxidant response elements in the nucleus, and stimulates the transcription of relevant antioxidant genes, such as HO-1, SOD, and CAT.^45^ Thus, we hypothesized that LPS stimulation could activate the Nrf2/HO-1 signaling pathway in HUVECs. Our results confirmed that this pathway was indeed activated in the LPS group and was subsequently downregulated following BA-BMSC-exos treatment (Figures S3A and S3B). These results indicate that BA-BMSC-exos may alleviate oxidative stress by modulating the Nrf2/HO-1 signaling pathway.

**Figure 4:**
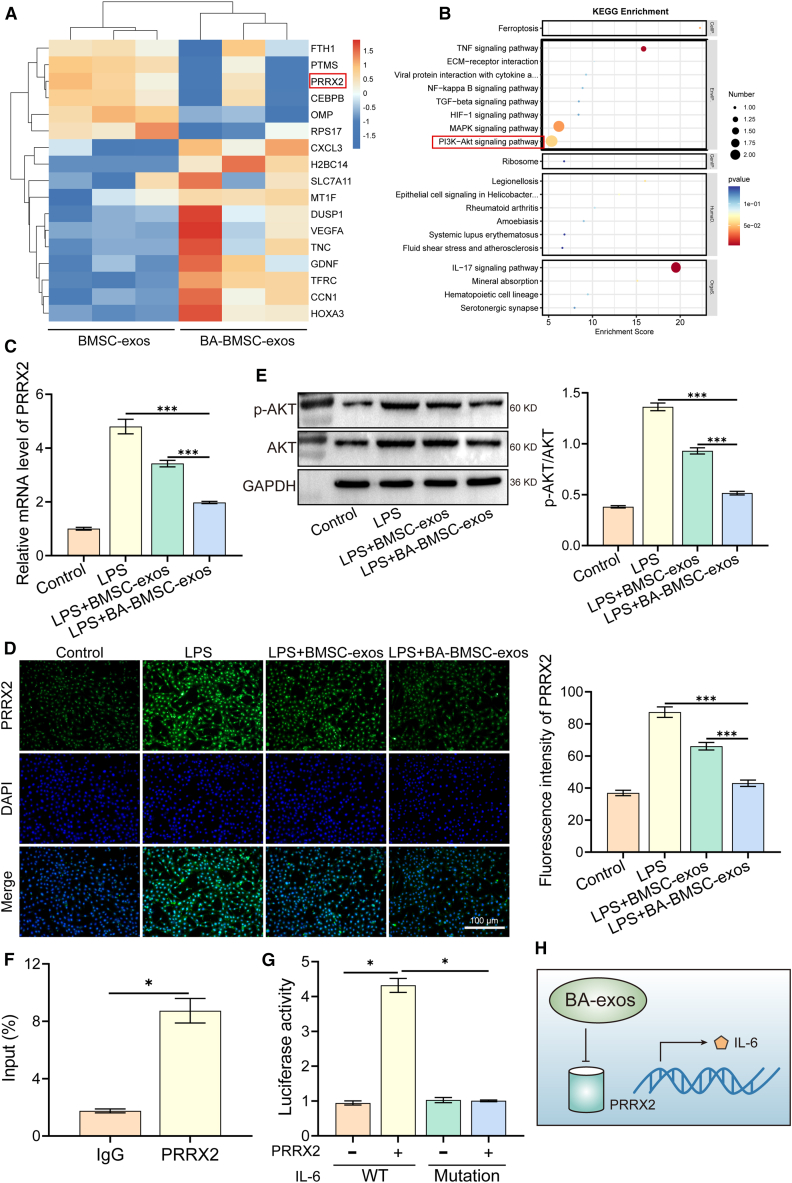
BA-BMSC-exos inhibited inflammation via PRRX2/IL-6/AKT pathway (A) The heatmap of RNA-seq data illustrates the differentially expressed genes. (B) KEGG pathway enrichment. (C) The mRNA level of PRRX2 in HUVECs. (D) Immunofluorescence analysis of PRRX2 in HUVECs (scale bar: 100 μm). (E) Western blot analysis of *p*-AKT in HUVECs. (F) The binding of Prrx2 to the IL-6 gene promoter was studied by using the ChIP method, and the promoter of IL-6 was amplified by qPCR. (G) Luciferase activity of wild or mutant IL-6 was tested by luciferase reporter assay. (H) Hypothetical scheme for the mechanisms of PRRX2 inhibiting inflammation. Data are presented as mean ± standard deviation (SD), *n* = 3, *p*-values are calculated using one-way ANOVA, ∗*p* < 0.05, ∗∗*p* < 0.01, ∗∗∗*p* < 0.001.

To further explore the possible molecular mechanism by which the BA-BMSC-exos inhibit inflammation. Heatmap revealed the distribution of differentially expressed genes in LPS-induced HUVECs (Figure 4A). It found that BA-BMSC-exos downregulated the expression of PRRX2, a transcription factor that has been implicated in the regulation of inflammatory responses.^46^ Furthermore, KEGG pathway analysis revealed that the PI3K-AKT signaling pathway was identified as one of the top twenty pathways significantly enriched with differentially expressed genes (Figure 4B). We further confirmed that BA-BMSC-exos significantly decreased the expression of PRRX2 compared to the LPS and LPS+BMSC-exos groups by qPCR analysis (Figure 4C). Immunofluorescence staining also demonstrated a weak PRRX2 expression in the LPS+BA-BMSC-exos group (Figure 4D). These findings demonstrated that BA-BMSC-exos could downregulate PRRX2. Furthermore, to further validate the involvement of the significantly enriched PI3K-AKT pathway, we assessed the phosphorylation level of AKT (pAKT). Western blot analysis displayed that BA-BMSC-exos treatment significantly reduced the protein expression of pAKT compared to the LPS+BMSC-exos group (Figure 4E). Although the results indicate that the PI3K-AKT pathway was activated in the LPS group, the regulatory relationship between AKT and PRRX2 remains to be elucidated, which can be further explored in future studies. To explore the potential mechanism by which PRRX2 modulates inflammation, we performed Chromatin Immunoprecipitation (ChIP) assays (Figure 4F), which showed that PRRX2 directly binds to the IL-6 promoter region, suggesting that PRRX2 regulates IL-6 expression at the transcriptional level. This finding was further validated using a dual-luciferase reporter assay (Figure 4G), which confirmed that PRRX2 regulates IL-6 expression through direct transcriptional activation. These findings highlight the mechanism through which BA-BMSC-exos modulate the inflammatory response by targeting key cytokine genes (Figure 4H). To further confirm that BA-BMSC-exos promote angiogenesis in HUVECs by modulating PRRX2, we performed rescue experiments by overexpressing PRRX2 (OE-PRRX2). Transwell assays demonstrated that OE-PRRX2 reduced the migration of HUVECs in the LPS+BA-BMSC-exos+OE-PRRX2 group (Figures S5B and S5C). qPCR analysis further showed that OE-PRRX2 downregulated the expression of VEGF and CD31 in LPS+BA-BMSC-exos+OE-PRRX2 group (Figure S5D). These results suggest that OE-PRRX2 can partially reverse the pro-angiogenic effects of BA-BMSC-exos. Recently, several studies have reported that exosomes derived from differently preconditioned cells can promote angiogenesis. Li et al. found that hypoxia preconditioned DPSC-derived exosomes regulate angiogenesis via transferring LOXL2.^47^ Yang et al. demonstrated that IL-1β-stimulated bone mesenchymal stem cell-derived exosomes enhanced the proliferation, migration, and tube formation of HUVECs, as well as promoted the polarization of M2 macrophages.^48^ Although these preconditioning strategies have shown beneficial effects on angiogenesis and immunomodulation, BA preconditioning offers distinct advantages due to its dual anti-inflammatory and antioxidant properties. This not only enhances the functional capacity of exosomes but also reduces cellular stress during exosome production.

### 3D-β-tricalcium phosphate@baicalin-pretreated bone mesenchymal stem cells exosomes-exos enhanced osteogenesis and angiogenesis *in vivo*

**Figure 5:**
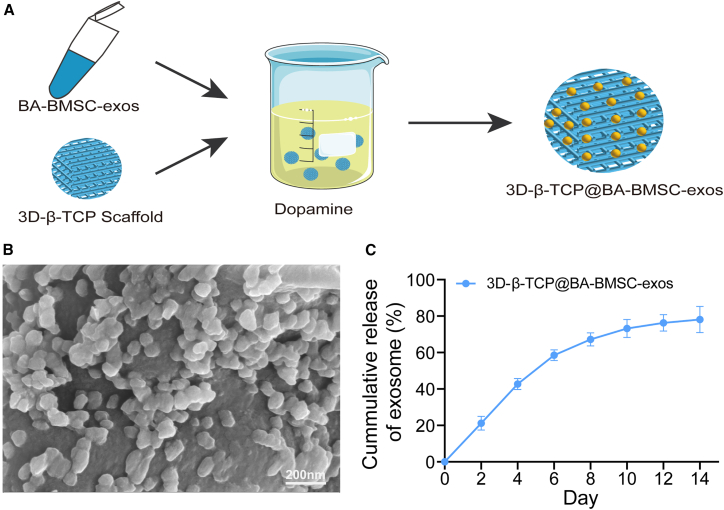
Characterization of 3D-β-TCP scaffold precoated with BA-BMSC-exos (A) Diagram showing the procedures of the incorporation of BA-BMSC-exos on 3D-β-TCP scaffolds. (B) SEM images of 3D-β-TCP@BA-BMSC-exos. (C) The release of BA-BMSC-exos from 3D-β-TCP scaffolds. Data are presented as mean ± standard deviation (SD), *n* = 3.

**Figure 6:**
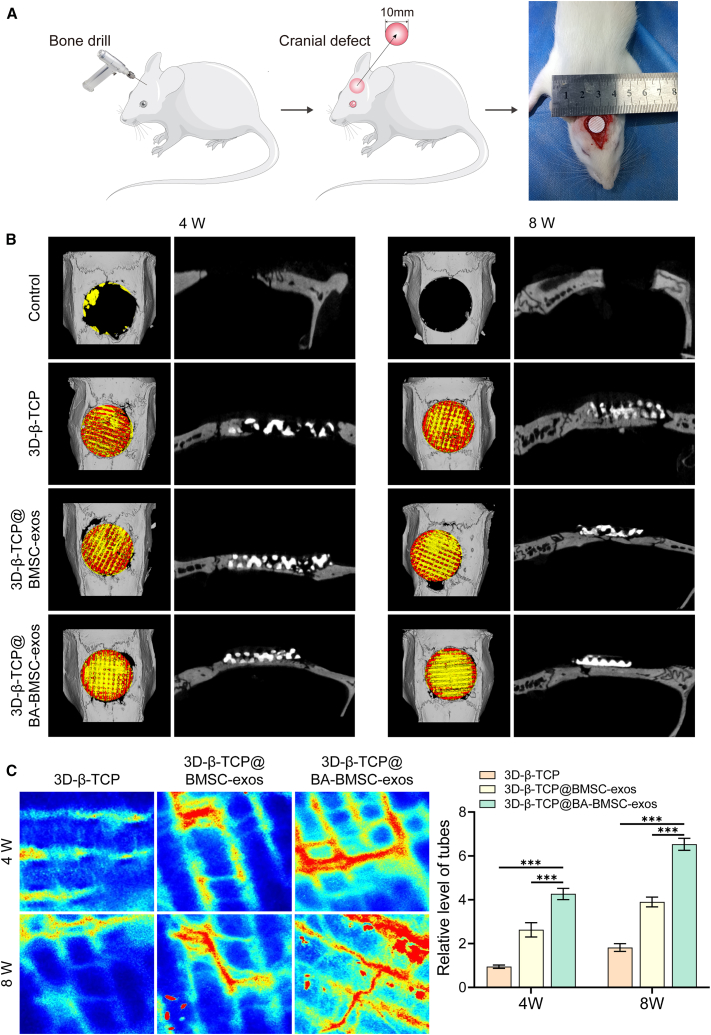
The effect of 3D-β-TCP@BA-BMSC-exos on bone regeneration in rat calvarial defects (A) Implantation procedure of scaffolds into the rat calvarial defect. (B) Micro-CT images of different groups on bone regeneration in rat calvarial defects at 4 and 8 weeks (C) Laser speckle images of scaffolds on bone regeneration in rat calvarial defects at 4 and 8 weeks. Data are presented as mean ± standard deviation (SD), *n* = 3, *p*-values are calculated using one-way or two-way ANOVA, ∗*p* < 0.05, ∗∗*p* < 0.01, ∗∗∗*p* < 0.001.

To assess the effect on osteogenesis and angiogenesis of 3D-β-TCP scaffolds loaded with BA-BMSC-exos *in vivo*, we used a rat calvarial defect model. Firstly, 3D-β-TCP scaffolds precoated with dopamine were incorporated with BA-BMSC-exos^49^ (Figure 5A). The morphology and distribution of BA-BMSC-exos loaded onto 3D-β-TCP scaffolds were analyzed using scanning electron microscopy (SEM). As shown in Figure 5B, BA-BMSC-exos were observed to be uniformly distributed throughout the scaffold material, indicating that BA-BMSC-exos were effectively loaded on the scaffold. Moreover, the BA-BMSC-exos were continuously released from the 3D-β-TCP scaffolds (Figure 5C). Then, the 3D-β-TCP scaffolds with or without exos were implanted into rats with calvarial defects and integrated into the native bone tissue with proper size and morphological matching (Figure 6A). Micro-CT imaging was performed to assess the osteogenic potential of 3D-β-TCP scaffolds (Figure 6B). The results showed that control and 3D-β-TCP groups limited the bone formation, with small amounts of new bone observed around the defect site. Otherwise, the groups treated with 3D-β-TCP@BMSC-exos and 3D-β-TCP@BA-BMSC-exos showed enhanced bone formation, with the 3D-β-TCP@BA-BMSC-exos group exhibiting the most substantial new bone formation and mineralization. Moreover, we found that 3D-β-TCP@BA-BMSC-exos could promote angiogenesis in calvarial tissues, as detected by laser speckle contrast imaging (Figure 6C). These results suggest that BA-BMSC-exos not only promote osteogenesis but also play a crucial role in angiogenesis, which is essential for successful tissue regeneration.

**Figure 7:**
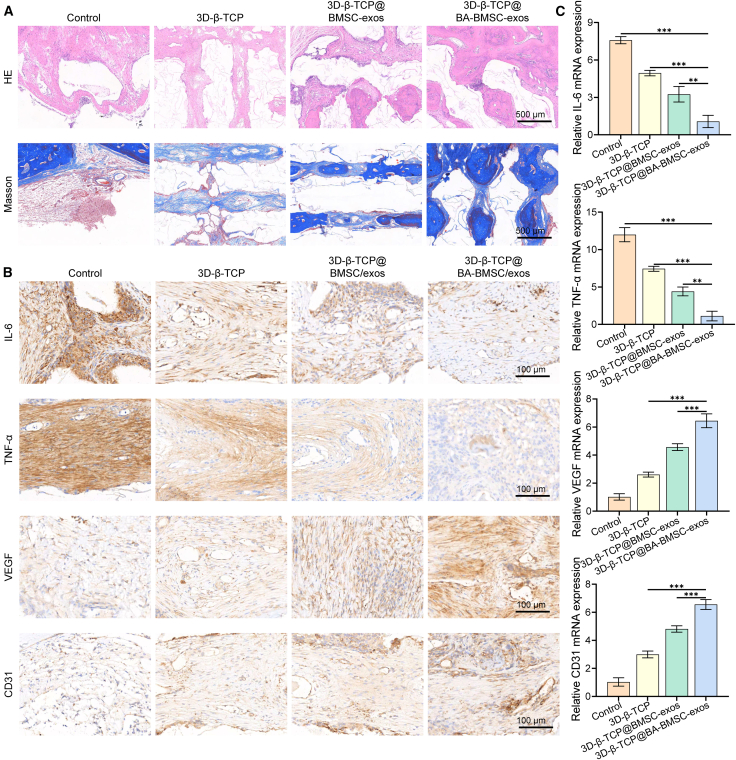
Effects of 3D-β-TCP scaffolds loaded with exos on angiogenesis and osteogenesis *in vivo* (A–C) HE staining and Masson staining analysis of the formation of new bone after implantation with a scaffold for 8 weeks (B and C) The expression levels of IL-6, TNF-α, VEGF, and CD31 were determined by immunohistochemistry staining and qPCR. Data are presented as mean ± standard deviation (SD), *n* = 3, *p*-values are calculated using one-way ANOVA, ∗*p* < 0.05, ∗∗*p* < 0.01, ∗∗∗*p* < 0.001.

Histological staining further confirmed the osteogenic and angiogenic effects of BA-BMSC-exos. HE staining revealed that 3D-β-TCP@BA-BMSC-exos group exhibited the most extensive formation of new bone and blood vessels compared to other groups after 8 weeks implantation (Figure 7A). Masson staining demonstrated collagen deposition, with more abundant and organized collagen fibers in the 3D-β-TCP@BA-BMSC-exos group, indicating enhanced osteogenesis (Figure 7A). Moreover, immunohistochemistry staining for markers of inflammatory cytokine (IL-6, TNF-α) and angiogenesis (CD31, VEGF) further confirmed the therapeutic effects of 3D-β-TCP@BA-BMSC-exos. As shown in Figure 7B, the 3D-β-TCP@BA-BMSC-exos group possessed a lower expression of IL-6 and TNF-α, suggesting the anti-inflammatory effect of BA-BMSC-exos. Additionally, the angiogenic markers CD31 and VEGF were more prominently expressed in 3D-β-TCP@BA-BMSC-exos group, indicating significant vascularization (Figure 7B). Additionally, qPCR (Figure 7C) and western blot analysis (Figure S4) were further supporting the role of BA-BMSC-exos in relieving inflammation and promoting angiogenesis. These results suggest that 3D-β-TCP@BA-BMSC-exos represent a promising strategy for enhancing osteogenesis and angiogenesis in bone tissue engineering.

In this study, we successfully developed a 3D-β-TCP scaffold loaded with BA-BMSC-exos derived from baicalin-pretreated bone mesenchymal stem cells (BMSCs) for the treatment of large bone defects. The results demonstrated that BA-BMSC-exos significantly promoted the proliferation, migration, and tube formation of HUVECs, while also inhibiting inflammation through the PRRX2/IL-6 signaling pathway. Furthermore, *in vivo* experiments using a calvarial defect rat model revealed that 3D-β-TCP scaffolds loaded with BA-BMSC-exos markedly enhanced the effects on anti-inflammatory and angiogenesis, leading to improved bone regeneration. These findings suggest that the combination of 3D-β-TCP scaffolds and BA-BMSC-exos holds great potential as a therapeutic strategy for enhancing bone repair and regeneration, offering a promising approach for the clinical management of bone defects.

### Limitations of the study

This study primarily focused on evaluating the biological efficacy of BA-BMSC-exos and the 3D-β-TCP@BA-BMSC-exos scaffold in promoting bone regeneration and modulating inflammation. However, several limitations remain. First, the long-term stability, storage conditions, and delivery logistics of the exosome-loaded scaffolds were not systematically investigated. Although previous studies have demonstrated that exosomes can retain their bioactivity when stored at −80°C or lyophilized under appropriate conditions, further research is required to establish standardized protocols suitable for clinical translation. Second, while the biodegradability of β-TCP has been well-documented in large animal models, such as pigs, where nearly complete degradation occurs over extended periods, this study did not perform a direct evaluation of scaffold degradation. Future work should include both *in vitro* and *in vivo* degradation assessments to determine how the degradation rate of the scaffold correlates with the pace of bone regeneration and tissue remodeling. Addressing these limitations will be essential for advancing the clinical applicability of the 3D-β-TCP@BA-BMSC-exos system.

## Resource availability

### Lead contact

Further information and requests for resources and reagents should be directed to and will be fulfilled by the lead contact, Huanwen Ding (dinghw@scut.edu.cn).

### Materials availability

This study did not generate new unique reagents or cell lines.

### Data and code availability

• All data supporting the findings of this study are found within the article and its supplemental information.
• This article does not report the original code.
• Any additional information about the data reported in this article will be shared by the lead contact upon request.

## Acknowledgments

This study was supported by the Guangzhou Key R&D Science and Technology Program (202206040001), the 10.13039/501100003453Natural Science Foundation of Guangdong Province (2023A1515010557), and the Science and Technology Planning Project of 10.13039/501100020757Guangzhou First People's Hospital in Guangdong Province, China (2023A03J0957).

## Author contributions

All authors participated in the conception, design, and implementation of the study. Haotian Zhu initially proposed the research idea and was responsible for the preliminary experimental planning. Under the guidance of Huanwen Ding, Naru Zhao, and Han Yan, Haotian Zhu finalized the research plan. Mingwei Tian and Yadi Zhang were responsible for the preparation and characterization of β-tricalcium phosphate scaffolds. Haotian Zhu, Kai Cheng, and Yuanhao Peng executed the cell and animal experiments and collected the data. Shaoxing Fan and Bo Shang were responsible for data organization and analysis. JiaYi Wu was responsible for animal feeding, daily maintenance, and literature review. Haotian Zhu drafted the initial article, and all authors contributed to the discussion, revision, and finalization of the article. The contributions of each author were crucial for the successful completion of this study.

## Declaration of interests

The authors declare that they have no competing interests.

## STAR★Methods

### Key resources table

**Table:** 

REAGENT or RESOURCE	SOURCE	IDENTIFIER
**Antibodies**		

CD9 Antibody	Abcam	Cat # ab236630; RRID: AB_2922400
TSG101 Antibody	Abcam	Cat # ab133586; RRID: AB_2943043
CD81 Antibody	Abcam	Cat # ab109201; RRID: AB_10866464
VEGF Antibody	Proteintech	Cat # 19003-1-AP; RRID: AB_2212657
CD31 Antibody	Proteintech	Cat # 66065-2-Ig; RRID: AB_2918476
TNF-α Antibody	Proteintech	Cat # 17590-1-AP; RRID:AB_2271853
IL-1β Antibody	Proteintech	Cat # 26048-1-AP; RRID:AB_2880351
IL-6 Antibody	Proteintech	Cat # 26404-1-AP; RRID:AB_3085866
AKT Antibody	Proteintech	Cat # 60203-2-Ig; RRID: AB_10912803
*p*-AKT Antibody	Proteintech	Cat # 66444-1-Ig; RRID: AB_2782958
NRF2 Antibody	Proteintech	Cat # 16396-1-AP; RRID: AB_2782956
HO-1 Antibody	Proteintech	Cat # 10701-1-AP; RRID: AB_2118685
GAPDH Antibody	Proteintech	Cat # 60004-1-Ig; RRID:AB_2107436

**Experimental models: Cell lines**		

BMSCs	Guangzhou Cyagen Biology	Cat # RAWMX-01001
HUVECs	Guangzhou Cyagen Biology	Cat # HUVEC-20001

**Experimental models: Organisms/strains**		

SD rats	Guangzhou Ruige Biotechnology Co., Ltd.	NA

**Software and algorithms**		

GraphPadPrism	GraphPadSoftware,LLC	V9.5
Fiji ImageJsoftware	ImageJ.org	V1.53

### Experimental model and study participant

#### Cell culture

BMSCs and HUVECs were purchased from Guangzhou Cyagen Biology (Cyagen, China), and cultured in mesenchymal stem cell medium (Cyagen, China) and endothelial cell growth medium-2 (ECM-2) (Lonza, Switzerland) with 10% FBS (Gibco, USA) and 1% penicillin–streptomycin (Gibco, USA), respectively. HUVECs were seeded onto the 3D-β-TCP scaffolds, and the cell viability and biocompatibility on scaffolds were analyzed using CCK8 assay kit and F-actin staining. Besides, HUVECs without LPS stimulation served as the control group, while the experimental groups were treated with 1 μg/mL LPS to simulate the bone defect environment *in vitro*, followed by treatment with BMSC-exos and BA-BMSC-exos before subsequent assays.

#### Animal experiment

Eight-week-old male SD rats (weighing 250 ± 10 g) were used in the animal experiments. The rats were randomly divided into 4 groups with 6 rats in each group: (1) control, (2) 3D-β-TCP scaffold, (3) 3D-β-TCP scaffold + BMSC-exos and (4) 3D-β-TCP scaffold + BA-BMSC-exos. A hole (10 mm in diameter) was drilled to establish the cranial defect model. The 3D-β-TCP scaffolds loaded with BA-BMSCS-exos matched with the defect were completely implanted. After suturing the skin and periosteum around the defect, the incision was closed and wrapped with gauze to prevent infection. After 4 and 8 weeks of implantation, tissue-scaffold constructs were collected and prepared for hematoxylin and eosin (HE), immunohistochemistry, qPCR and western blot tests. The animal experiments were approved by the Animal Ethics Committee of South China University of Technology.

### Method details

#### Preparation of 3D-Printed β-TCP scaffolds

β-TCP scaffolds were fabricated using 3D printing technology. β-TCP powder was synthesized via a solid-state reaction, surface-modified, and mixed with acrylic resin to create the printing paste. The scaffold model was designed and sliced into layers using SDPSlicer software (Ten-Dimension Tech Co., Ltd., Shenzhen, China), with a layer thickness of 50 μm. The paste was printed using a Ten-Dimension 3D printer, curing each layer under UV light. After printing, scaffolds were cleaned with ultrasound and dried for 24 h. The final scaffold was calcined at 1100 °C to form a ceramic structure. The scaffold dimensions were verified using CT scanning for 3D reconstruction.

#### Isolation and characterization of the BMSC-exos

The method used for exos isolation was performed as previously reported.^50^ BMSCs at a density of 1×10^6^ cells were cultured in DMEM/F12 medium containing 10% fetal bovine serum and 1% antibiotics until reaching a cell density of 70–80%, and then treated with 10 μM Baicalin for 24 h. The culture medium of BA-pretreated BMSCs was collected and centrifuged at 300×g for 10 min and then at 2000×g for 10 min at 4 °C to obtain the supernatants. Next, the supernatants were centrifuged for 30 min at 10,000×g and then filtered through a 0.22 μm sterilized filter. Subsequently, the supernatant was ultracentrifuged at 140,000×g for 70 min to harvest the exos. The resulting pellet was further purified by resuspension in PBS for subsequent use. Protein concentration was determined using a BCA protein assay kit (Seyotin, China). Exos were identified and examined through nanoparticle tracking analysis (NTA, Particle-Metrix, GA) and transmission electron microscopy (TEM, Hitachi, Japan).

#### Exos uptake assay

HUVECs were seeded in confocal dishes and incubated overnight. Exos were fluorescently labeled with PKH67 (Sigma, USA) and incubated with hepatocytes for 24 h. The uptake of exosomes was observed under a laser confocal microscope (Carl Zeiss, Germany).

#### CCK-8 assay

A CCK-8 assay kit (Seyotin, China) was used to assess the cell viability on HUVECs. HUVECs were cultured in 96-well plates at a density of 1×10^4^ cells/well and then treated with different interventions for 48 h. Next, 10 μL of CCK-8 solution was added to each well and the incubation was continued at 37 °C for 2 h. The OD values were measured at 450 nm.

#### EdU staining assay

5-ethynyl-2′-deoxyuridine (EdU, Beyotime, China)) was used to measure cell proliferation. HUVECs were initially seeded in 24-well plates at a concentration of 1×10^4^ cells per well and cultured for 24 h. Then, the cells in each well were incubated with 100 μL EdU working solution for 2 h at 37 °C. After washing with PBS for 5 min twice, cells were fixed with 4% neutral paraformaldehyde for 25 min and then permeabilized with 0.5% Triton X-100 for 10 min. Finally, cells were stained with 100 μL of Appllo solution at room temperature in the dark for 30 min, and then incubated with 100 μL of Hoechst 33342 solution in the dark for 30 min. The respective images were acquired by a confocal microscope.

#### Transwell migration and tube formation assay

For Transwell migration: HUVECs (1×10^5^) were resuspended in serum-free medium and then seeded into the upper chambers of transwells (Corning, USA). 600 μL medium with or without exos were added to the bottom chamber. After incubation for 24 h, the cells were transferred to the bottom of the membrane and then fixed with methanol for 30 min. The migrated cells were stained using 1% crystal violet for 20 min and imaged via an inverted microscopy.

For tube migration: HUVECs were treated with 10 μg/mL BMSC-exos and BA-BMSC-exos, and seeded into 24-well plates coated with Matrigel at a density of 1×10^5^ cells/well. After incubation for 24 h, the number of tubes was counted under an optical microscope.

#### Western blot assay

Proteins from cells, exosomes, and tissues were harvested and subjected to SDS/PAGE electrophoresis for 90 min. Following separation, the proteins were transferred to PVDF membranes on ice for 90 min. The membranes were then incubated with a blocking solution containing 5% BSA for 1 h to prevent non-specific binding. After washing three times, the membranes were stained with primary antibodies against CD9, TSG101, CD31, *p*-AKT, AKT, IL-6, IL-1β, TNF-α, Nrf2, and HO-1 (all from Abcam, UK), vascular endothelial growth factor (VEGF) (from Proteintech, USA) overnight at 4 °C. Subsequent to three additional washes, the membranes were incubated with the secondary antibody for 1 h at room temperature. After washing the membrane again with TBST three times, the membrane was developed using ECL reagent. The protein expression levels were subsequently quantified by ImageJ software.

#### qPCR

Total RNA was extracted from the liver using Trizol reagent (Takara, Japan). cDNA was synthesized, and qPCR was performed. The relative expression of the target gene was normalized against GAPDH and analyzed using the 2^−ΔΔCt^ method. The primer sequences are shown in Table S1.

#### Flow cytometry assay

HUVECs (1×10^5^ cells/well) were seeded in 6-well plates for 16 h, followed by different treatments. Later, cells were washed with PBS and harvested by trypsin treatment. The cells were then resuspended with 0.5 mL PBS containing 10 μM of 2′, 7′-dichlorodihydro-fluorescein diacetate (DCFDA, Invitrogen) fluorescent probe for 30 min. The cells were collected and immediately analyzed by a flow cytometer.

#### Dual-luciferase assay

The Dual-Luciferase Reporter Assay was performed utilizing the Luciferase Detection Kit (KeyGEN BioTECH, China). Firstly, a luciferase reporter plasmid driven by the promoter region of PRRX2 was constructed by inserting the promoter region of PRRX2 into the pGL3-Basic vector by subcloning. This reporter plasmid was co-transfected into cells with an expression plasmid containing PRRX2 or a corresponding control sequence. After transfection, cell lysates were prepared by adding reporter lysis buffer. Subsequently, 100 μL of the working solution was mixed with 20 μL of the sample, and luciferase activity was measured using a multifunctional enzyme labeling instrument (TECAN, Switzerland). The ratio of firefly luciferase activity to renilla luciferase activity was calculated to indicate the relative luciferase activity.

#### Chromatin immunoprecipitation (ChIP) assay

ChIP Detection Kit (Wanleibio, China) was used for ChIP assays according to the manufacturer’s protocol. Cells were treated with 1% formaldehyde to cross-link histones to DNA. The cells were harvested in SDS lysis buffer and then digested by proteinase inhibitor. Cell lysates were sonicated to shear DNA. Sheared chromatin was precleared with protein G beads prior to incubation overnight at 4 °C with anti-PRRX2 antibody or control IgG. Purified and immunoprecipitated chromatin fragments from IP samples were subjected to PCR. PCR amplification of the IL-6 gene promoter was performed with primers of the IL-6 promoter. PCR products were subjected to 2% agarose gel electrophoresis and stained with ethidium bromide.

#### Laser speckle contrast imaging

Rats were anesthetized with an injection of 5% chloral hydrate and secured on a stereotaxic frame. After shaving and disinfecting the scalp, the skull surface was exposed to image. A laser was directed onto the skull to capture speckle images. The raw speckle images were then processed by the system to generate blood flow images, and cerebral blood flow over the skull surface was calculated through speckle contrast analysis.

#### Microcomputed tomography evaluation

The specimens were subjected to micro-CT scanning (Bruker SkyScan 1276, Billerica, MA, USA; 85 kV, 200 mA, 10 μm resolution). The three-dimensional reconstruction of the sagittal and axial sections was performed using the Data Viewer software (Version 1.5.2.4; Bruker, Billerica, MA, USA).

#### Histological analysis

After Micro-CT scanning, the fixed craniums tissue was decalcified with EDTA solution, gently shaken for up to 2 weeks, then dehydrated, transparentized, and sliced. H&E, and Masson were carried out and imaged by microscope. Immunofluorescence staining was performed as follows. The cells and slices of tissues were fixed in 4% paraformaldehyde for 30 min at room temperature. After being soaked with PBS 3 times, the cells and slices were blocked with normal goat serum at room temperature for 30 min. Then, the cells and slices were incubated with primary antibodies at 4 °C for 8–16 h. After being soaked with PBS three times, the cells and slices were stained with secondary antibodies in the dark for 1 h. The nuclei were stained with DAPI in the dark for 5 min. The respective images were acquired by a confocal microscope (Carl Zeiss, Germany).

### Quantification and statistical analysis

Statistical analyses were performed using GraphPad Prism 9.5 software. Data presents a mean ± standard deviation. One-way analysis of variance (ANOVA) or two-way analysis of variance were used to assess statistical significance. A ∗*p* value of ≤0.05 was deemed statistically significant, ∗∗*p* ≤ 0.01 was regarded as moderately significant, and ∗∗∗*p* ≤ 0.001 was considered highly statistically significant.
