# Supplementary material for: 3D-printed scaffold loaded with baicalin exosomes promotes bone defect repair via mediating PRRX2 to alleviate inflammation
Source: iScience. 2025 Sep 12;29(1):113565. doi: 10.1016/j.isci.2025.113565 (PMC12796540; doi:10.1016/j.isci.2025.113565)
Supplement: Document S1. Figures S1–S5 and Table S1 [file mmc1.pdf]

iScience, Volume *29*

Supplemental information

3D-printed scaffold loaded with baicalin exosomes promotes bone defect repair via mediating PRRX2 to alleviate inflammation

Haotian Zhu, Kai Cheng, Mingwei Tian, Yuanhao Peng, Yadi Zhang, Han Yan, Shaoxing Fan, Bo Shang, JiaYi Wu, Huanwen Ding, and Naru Zhao

Supporting information

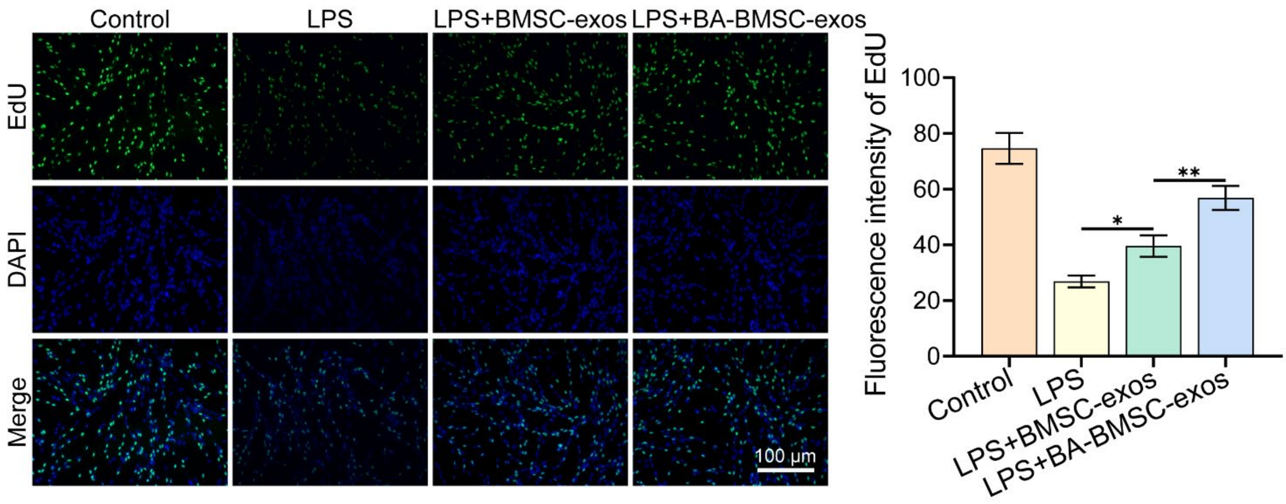

Fig S1. Effect of BA-BMSC-exos on the proliferation of HUVECs. EdU analysis of different groups in LPS-induced HUVECs (scale bar: 100 μm). Data are presented as mean ± standard deviation (SD), n=3, *P*-values are calculated using one-way ANOVA, **P* < 0.05, ***P* < 0.01, ****P* < 0.001.

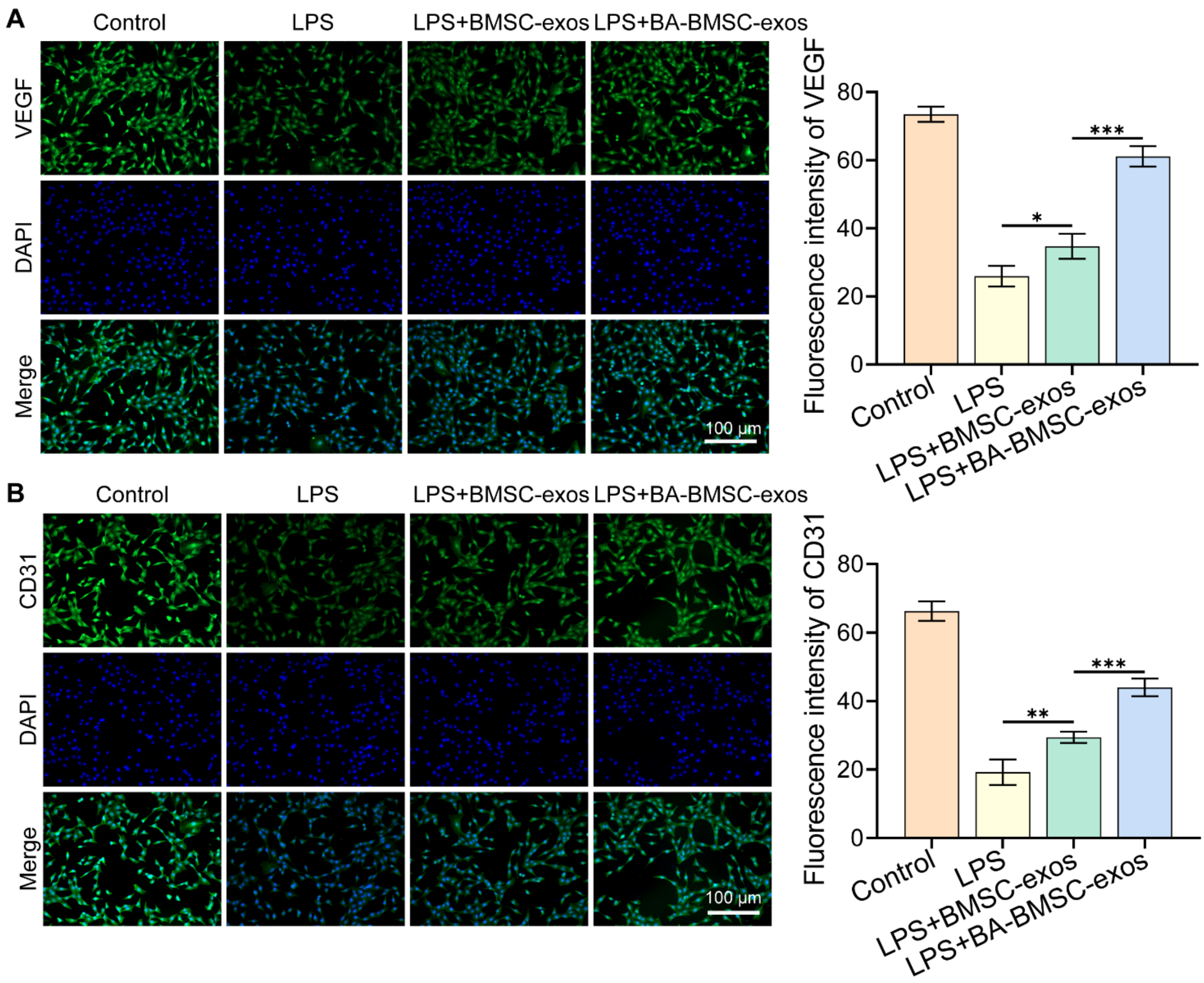

Fig S2. Angiogenesis of BA-BMSC-exos. (A and B) Immunofluorescence analysis of the expression of VEGF and CD31 LPS-induced HUVECs (scale bar: 100 μm). Data are presented as mean ± standard deviation (SD), n=3, *P*-values are calculated using one-way ANOVA, $^{*}P < 0.05$, $^{**}P < 0.01$, $^{***}P < 0.001$.

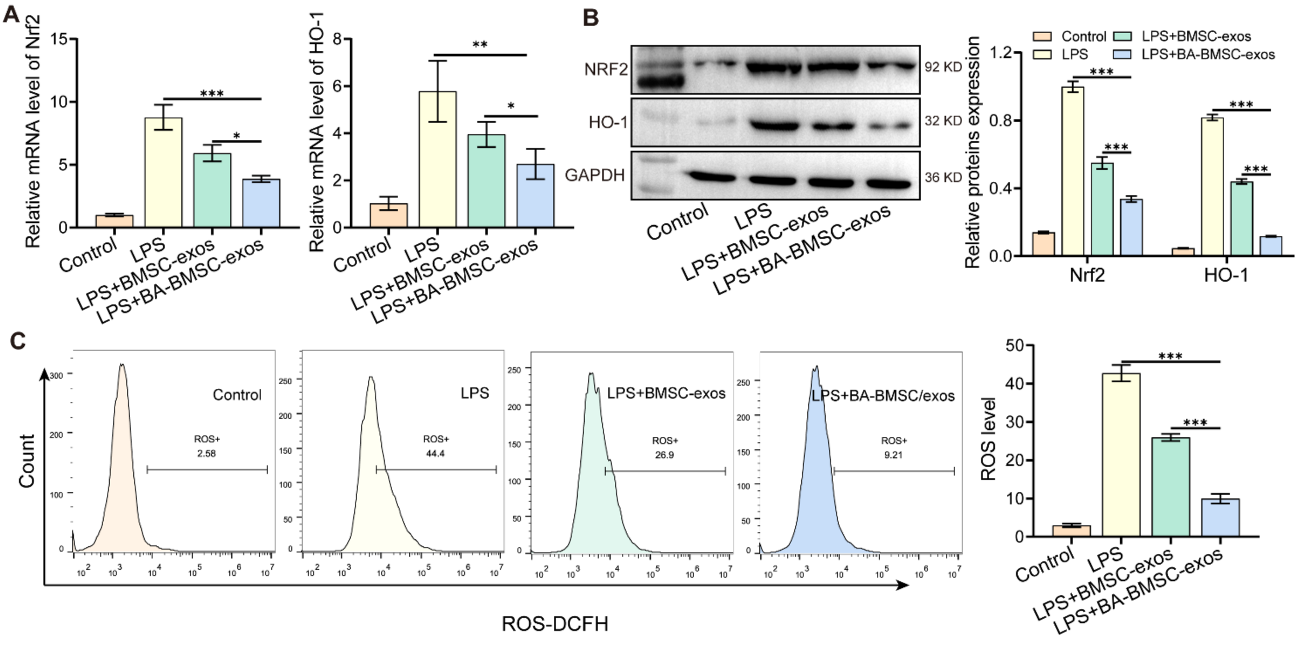

Fig S3. Anti-inflammation of BA-BMSC-exos. (A) The mRNA expression levels of Nrf2 and HO-1. (B) The protein expression levels of Nrf2 and HO-1. (C) Flow cytometry analysis of the ROS level. Data are presented as mean ± standard deviation (SD), n=3, *P*-values are calculated using one-way ANOVA, $^{*}P < 0.05$, $^{**}P < 0.01$, $^{***}P < 0.001$.

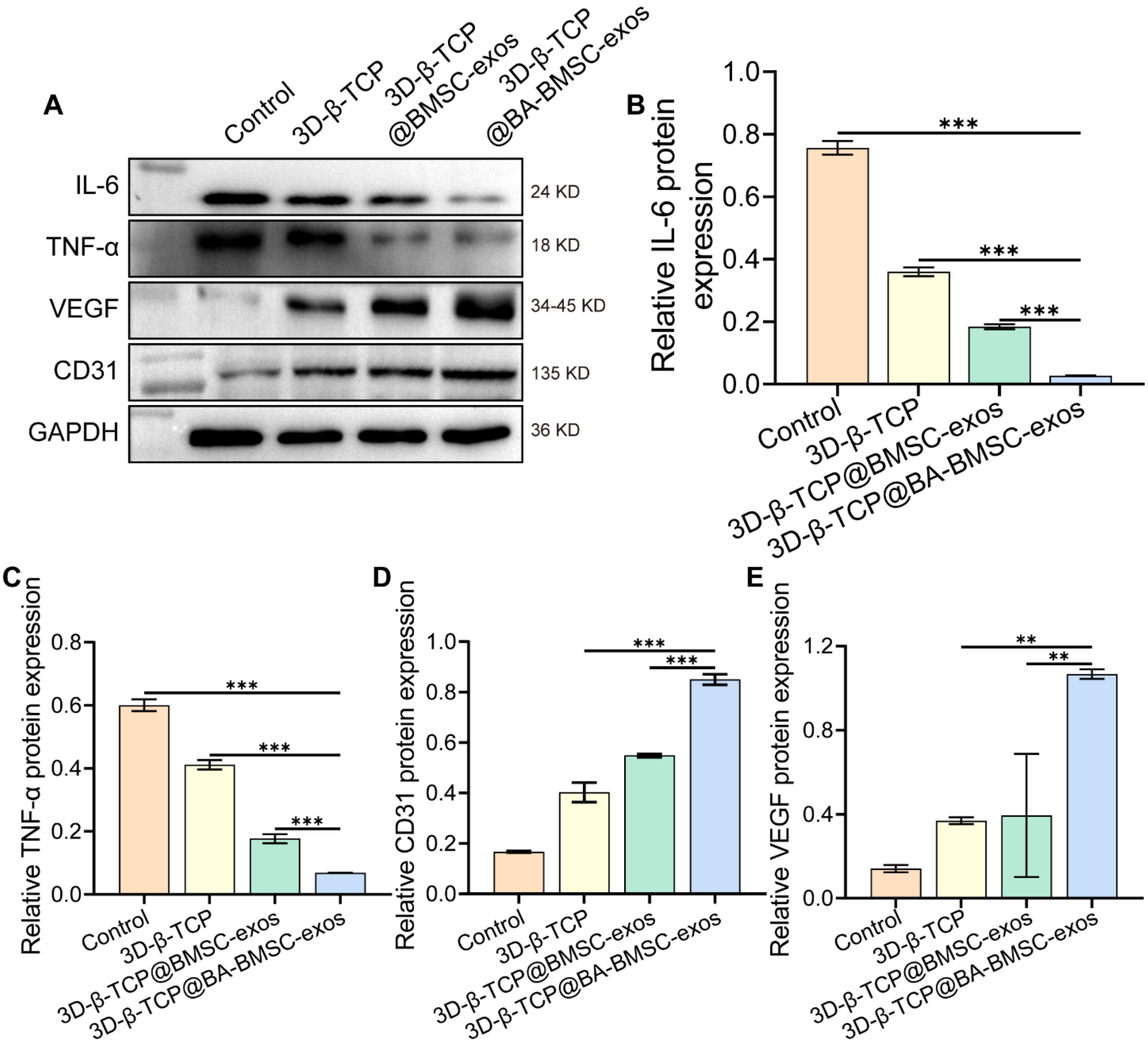

Fig S4. Effects of 3D-β-TCP scaffolds loaded with exos on inflammation. Western blot analysis of the expression levels of IL-6, TNF-α, VEGF, and CD31 on bone regeneration in rat calvarial defects. Data are presented as mean ± standard deviation (SD), n=3, *P*-values are calculated using one-way ANOVA, *$P < 0.05$, **$P < 0.01$, ***$P < 0.001$.

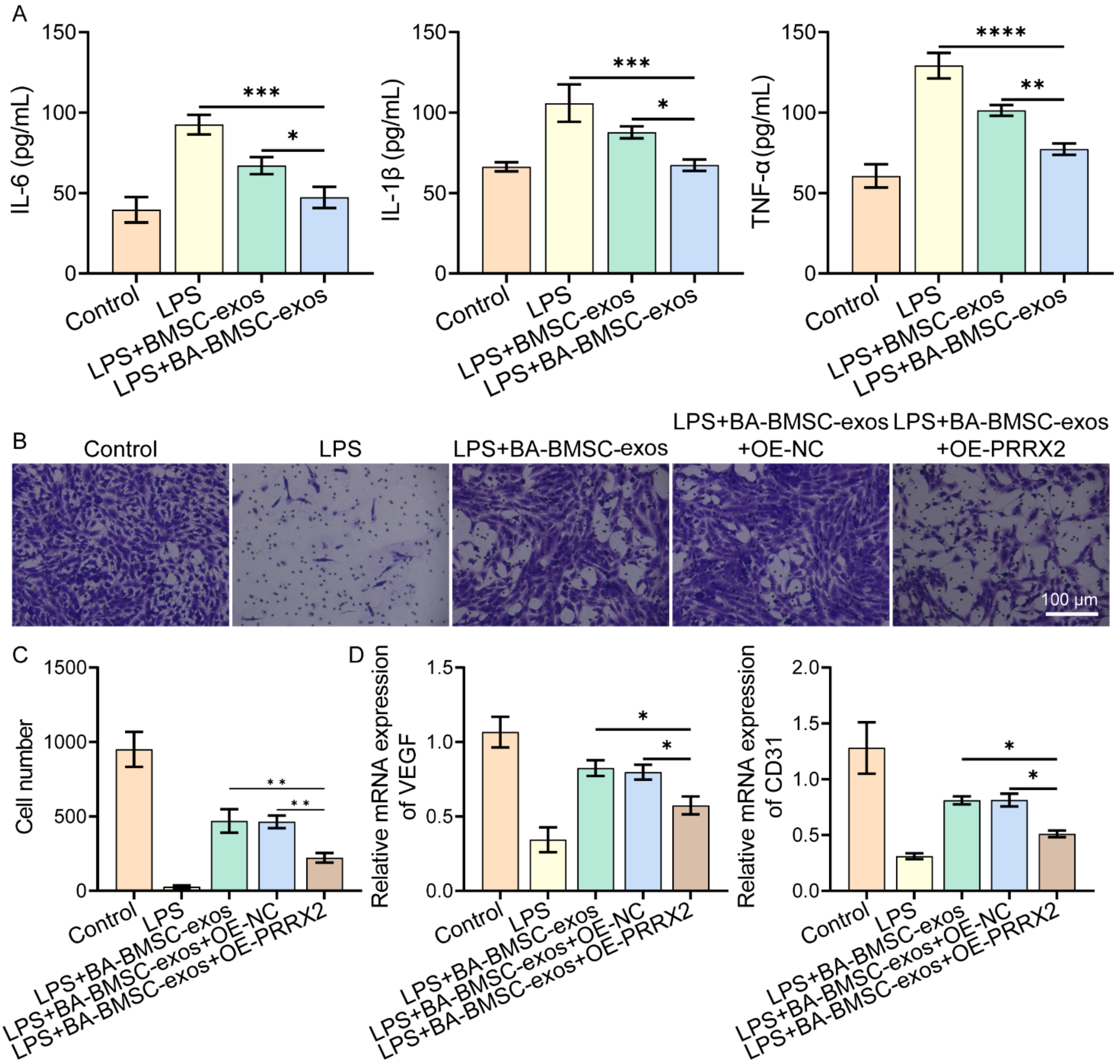

Fig S5. BA-BMSC-exos inhibited inflammation via PRRX2. (A) ELISA analysis of the levels of IL-6, IL-1β, and TNF-α in LPS-induced HUVECs after 48 h treatment. (B-C) Cell migration of HUVECs determined by Transwell assay (scale bar: 100 μm). (D) qPCR analysis of the mRNA expression of VEGF and CD31 in LPS-induced HUVECs. Data are presented as mean ± standard deviation (SD), n=3, *P*-values are calculated using one-way ANOVA, $*P < 0.05$, $**P < 0.01$, $***P < 0.001$.

Table S1. Primer sequences

Human

Genes	Forward (5'-3')	Reverse (5'-3')
VEGF	CCAAGGCCAGCACATAGGAG	TTGCGCTTTCGTTTTTGCCC
CD31	AGGAACAGGACCGCGTTTTA	CCATGATCATTCCGGTGGGG
IL-1β	CCCACAGACCTTCCAGGAGA	GCGTGCAGTTCAGTGATCGT
IL-6	GGCACTGGCAGAAAACAACC	CACAGCTCTGGCTTGTTCCT
TNF-α	TCTTCTGCCTGCTGCACTTT	GGGTTTGCTACAACATGGGC
PRRX2	CTTTGTGCGCGAGGAGCTTG	GCCAGCATGGCCCTTTCAT
NRF2	TCCCAGGTTGCCCACATTC	ACTGGGCTCTCGATGTGACC
HO-1	CACCCAGGCAGAGAATGCT	CGAAGACTGGGCTCTCCTTG
GAPDH	GCGCTGAGTACGTCGTGGA	AACATGGGGGCATCAGCAG

Mouse

Genes	Forward (5'-3')	Reverse (5'-3')
IL-6	CACTTCACAAGTCGGAGGCT	TTTCTGCAAGTGCATCATCGT
TNF-α	CACCACGCTCTTCTGTCTACT	CTCCTCCACTTGGTGGTTTGT
VEGF	CTCCACCATGCCAAGTGGTC	CCAGGGTCTCAATTGGACGG
CD31	GCACCGTGATACTGAACAGC	TGACTGTCACAATCCCACCTTC
GAPDH	CTTCCGTGTTCCTACCCCCAA	ATGCCCTTTAGTGGGCCCTC
